# Supplementary material for: Choosing to succeed? Insights into doctoral students’ supervisor selection and its outcomes
Source: PLoS One. 2025 Jul 21;20(7):e0328471. doi: 10.1371/journal.pone.0328471 (PMC12279141; doi:10.1371/journal.pone.0328471)
Supplement: Attachment — Submitted filename: PONE-D-24-33457_reviewer (1).pdf [file pone.0328471.s001.pdf]

PLOS ONE

Choosing to succeed? Insights into doctoral students’ supervisor selection and its outcomes

--Manuscript Draft--

**Manuscript Number:**	PONE-D-24-33457
**Article Type:**	Research Article
**Full Title:**	Choosing to succeed? Insights into doctoral students’ supervisor selection and its outcomes
**Short Title:**	Insights into doctoral students’ supervisor selection and its outcomes
**Corresponding Author:**	Danila Pavliuk<br>HSE University: Nacional'nyj issledovatel'skij universitet Vyssaa skola ekonomiki<br>Moscow, RUSSIAN FEDERATION
**Keywords:**	doctoral education; doctoral students; doctoral supervision; supervisor selection
**Abstract:**	Doctoral student experience and outcomes largely depend on the quality of supervision. Research usually focuses on the attributes, styles and roles of a supervisor during doctoral training, while the process and effects of initiating supervisor-supervisee relationships remain less explored. This paper aims to examine how students’ strategies of supervisor selection are related to consequent difficulties in interaction with supervisors and students’ confidence in a future dissertation defense. Based on a nationwide survey of Russian doctoral students (N=1796) and regression analysis, we demonstrate that more academic criteria such as supervisor’s subject-matter expertise and successful supervision history are the most optimal in terms of reducing probability to face difficulties with supervisor in future and students’ lack of confidence. Positive personal relationships with a supervisor as a criterion only reduce the chances to face interaction difficulties, but do not affect students’ confidence in future academic success. At the same time, the “only option” scenario, i.e., a situation when students have no alternatives when selecting a supervisor, is associated with higher chances to face difficulties during doctoral journey and lower level of students’ confidence. The results of the research can be used by prospective doctoral students, as well as doctoral program administration to create a personal or institutional navigation system for an effective choice of a supervisor.
**Order of Authors:**	Danila Pavliuk
	Svetlana Zhuchkova
**Additional Information:**	
**Question**	**Response**
**Financial Disclosure**<br><br>Enter a financial disclosure statement that describes the sources of funding for the work included in this submission. Review the submission guidelines for detailed requirements. View published research articles from *PLOS ONE* for specific examples.<br><br>This statement is required for submission and **will appear in the published article** if the submission is accepted. Please make sure it is accurate.	Yes

Powered by Editorial Manager® and ProduXion Manager® from Aries Systems Corporation

**Funded studies**<br>Enter a statement with the following details:<br>• Initials of the authors who received each award<br>• Grant numbers awarded to each author<br>• The full name of each funder<br>• URL of each funder website<br>• Did the sponsors or funders play any role in the study design, data collection and analysis, decision to publish, or preparation of the manuscript?<br><br>Did you receive funding for this work?	
Please add funding details.<br>as follow-up to "**Financial Disclosure**<br><br>Enter a financial disclosure statement that describes the sources of funding for the work included in this submission. Review the submission guidelines for detailed requirements. View published research articles from *PLOS ONE* for specific examples.<br><br>This statement is required for submission and **will appear in the published article** if the submission is accepted. Please make sure it is accurate.<br><br>**Funded studies**<br>Enter a statement with the following details:<br>• Initials of the authors who received each award<br>• Grant numbers awarded to each author<br>• The full name of each funder<br>• URL of each funder website<br>• Did the sponsors or funders play any role in the study design, data collection and analysis, decision to publish, or preparation of the manuscript?<br><br>Did you receive funding for this work?"	Both DP and SZ received support from the project "International academic cooperation" at HSE University. The funder had no role in study design, data collection and analysis, decision to publish, or manuscript preparation.
Please select the country of your main research funder (please select carefully as in some cases this is used in fee calculation).	RUSSIAN FEDERATION - RU

as follow-up to "**Financial Disclosure**<br><br>Enter a financial disclosure statement that describes the sources of funding for the work included in this submission. Review the submission guidelines for detailed requirements. View published research articles from *PLOS ONE* for specific examples.<br><br>This statement is required for submission and **will appear in the published article** if the submission is accepted. Please make sure it is accurate.<br><br>**Funded studies**<br>Enter a statement with the following details:<br>• Initials of the authors who received each award<br>• Grant numbers awarded to each author<br>• The full name of each funder<br>• URL of each funder website<br>• Did the sponsors or funders play any role in the study design, data collection and analysis, decision to publish, or preparation of the manuscript?<br><br>Did you receive funding for this work?"	
**Competing Interests**<br><br>Use the instructions below to enter a competing interest statement for this submission. On behalf of all authors, disclose any competing interests that could be perceived to bias this work—acknowledging all financial support and any other relevant financial or non-financial competing interests.<br><br>This statement is required for submission and **will appear in the published article** if the submission is accepted. Please make sure it is accurate and that any funding sources listed in your Funding Information later in the submission form are also declared in your Financial Disclosure statement.	The authors have declared that no competing interests exist.

Powered by Editorial Manager® and ProduXion Manager® from Aries Systems Corporation

View published research articles from *PLOS ONE* for specific examples.<br><br>**NO authors have competing interests**<br><br>Enter: *The authors have declared that no competing interests exist.*<br><br>**Authors with competing interests**<br><br>Enter competing interest details beginning with this statement:<br><br>*I have read the journal's policy and the authors of this manuscript have the following competing interests: [insert competing interests here]*<br><br>* typeset	
**Ethics Statement**<br><br>Enter an ethics statement for this submission. This statement is required if the study involved:<br><br>• Human participants<br>• Human specimens or tissue<br>• Vertebrate animals or cephalopods<br>• Vertebrate embryos or tissues<br>• Field research<br><br>Write "N/A" if the submission does not require an ethics statement.<br><br>General guidance is provided below. Consult the submission guidelines for detailed instructions. **Make sure that all information entered here is included in the Methods section of the manuscript.**	The study was reviewed and approved by the Institutional Review Board at HSE University. Statement of Opinion on compliance of the Empirical Research Project with ethical norms: "The empirical research project conforms entirely to ethical standards and can be implemented in its current form".

Powered by Editorial Manager® and ProduXion Manager® from Aries Systems Corporation

**Format for specific study types**<br><br>**Human Subject Research (involving human participants and/or tissue)**<br>• Give the name of the institutional review board or ethics committee that approved the study<br>• Include the approval number and/or a statement indicating approval of this research<br>• Indicate the form of consent obtained (written/oral) or the reason that consent was not obtained (e.g. the data were analyzed anonymously)<br><br>**Animal Research (involving vertebrate animals, embryos or tissues)**<br>• Provide the name of the Institutional Animal Care and Use Committee (IACUC) or other relevant ethics board that reviewed the study protocol, and indicate whether they approved this research or granted a formal waiver of ethical approval<br>• Include an approval number if one was obtained<br>• If the study involved *non-human primates*, add *additional details* about animal welfare and steps taken to ameliorate suffering<br>• If anesthesia, euthanasia, or any kind of animal sacrifice is part of the study, include briefly which substances and/or methods were applied<br><br>**Field Research**<br><br>Include the following details if this study involves the collection of plant, animal, or other materials from a natural setting:<br>• Field permit number<br>• Name of the institution or relevant body that granted permission	
**Data Availability**<br><br>Authors are required to make all data underlying the findings described fully available, without restriction, and from the time of publication. PLOS allows rare exceptions to address legal and ethical concerns. See the PLOS Data Policy and FAQ for detailed information.	Yes - all data are fully available without restriction

A Data Availability Statement describing where the data can be found is required at submission. Your answers to this question constitute the Data Availability Statement and **will be published in the article**, if accepted.<br><br>**Important:** Stating 'data available on request from the author' is not sufficient. If your data are only available upon request, select 'No' for the first question and explain your exceptional situation in the text box.<br><br>Do the authors confirm that all data underlying the findings described in their manuscript are fully available without restriction?	
**Describe where the data may be found in full sentences. If you are copying our sample text, replace any instances of XXX with the appropriate details.**<br><br>• If the data are **held or will be held in a public repository**, include URLs, accession numbers or DOIs. If this information will only be available after acceptance, indicate this by ticking the box below. For example: *All XXX files are available from the XXX database (accession number(s) XXX, XXX.).*<br>• If the data are all contained **within the manuscript and/or Supporting Information files**, enter the following: *All relevant data are within the manuscript and its Supporting Information files.*<br>• If neither of these applies but you are able to provide **details of access elsewhere**, with or without limitations, please do so. For example:<br><br>*Data cannot be shared publicly because of [XXX]. Data are available from the XXX Institutional Data Access / Ethics Committee (contact via XXX) for researchers who meet the criteria for access to confidential data.*<br><br>*The data underlying the results presented in the study are available from (include the name of the third party*	We have published regression dataset that used in our research at Harvard Dataverse under CC0 v1.<br><br>Pavliuk, Danila; Zhuchkova, Svetlana, 2024, "Replication Data for research paper: Choosing to succeed? Insights into doctoral students' supervisor selection and its outcomes", https://doi.org/10.7910/DVN/YKNXO3, Harvard Dataverse, V1

and contact information or URL).<br>• This text is appropriate if the data are owned by a third party and authors do not have permission to share the data.<br><br>* typeset	
Additional data availability information:	

The file with the manuscript
Click here to access/download;Manuscript;manuscript_dp_sz.docx

Choosing to succeed? Insights into doctoral students' supervisor selection and its outcomes

Danila Pavliuk (1), Svetlana Zhuchkova (2)

(1) Research Assistant, Center for Sociology of Higher Education, HSE University; *dmpavlyuk@hse.ru*

(2) Research Fellow, Center for Sociology of Higher Education, HSE University; *szhuchkova@hse.ru*

Corresponding author E-mail: dmpavlyuk@hse.ru

Abstract

Doctoral student experience and outcomes largely depend on the quality of supervision. Research usually focuses on the attributes, styles and roles of a supervisor during doctoral training, while the process and effects of initiating supervisor-supervisee relationships remain less explored. This paper aims to examine how students' strategies of supervisor selection are related to consequent difficulties in interaction with supervisors and students' confidence in a future dissertation defense. Based on a nationwide survey of Russian doctoral students (N=1796) and regression analysis, we demonstrate that more academic criteria such as supervisor's subject-matter expertise and successful supervision history are the most optimal in terms of reducing probability to face difficulties with supervisor in future and students' lack of confidence. Positive personal relationships with a supervisor as a criterion only reduce the chances to face interaction difficulties, but do not affect students' confidence in future academic success. At the same time, the "only option" scenario, i.e., a situation when students have no alternatives when selecting a supervisor, is associated with higher chances to face difficulties during doctoral journey and lower level of students' confidence. The results of the research can be used by prospective doctoral students, as well as doctoral program

administration to create a personal or institutional navigation system for an effective choice of a supervisor.

Keywords: doctoral education, doctoral students, doctoral supervision, supervisor selection

Introduction

"Supervision is the most variable of all variables" [1], as Bowen and Rudenstine wrote in the early 90s. Indeed, supervision is repeatedly considered a key component in the success of a doctoral student [2–4]. Research demonstrates that positive supervision experiences increase the satisfaction level, enhance research progress, and maintain the psychological well-being of doctoral students [5,6]. Conversely, imbalanced and inadequate academic advising may have reverse effects, degrading students' experience [7]. Such various outcomes have led national education systems and the academic community to become keenly interested in enhancing the quality of supervision.

Previous research in this area has focused on the styles, functions, and roles of academic supervisors in a doctoral journey [4,8,9]. These studies have primarily examined student-supervisor interactions during doctoral training, but there is scant literature on prior relationships between them, including how supervisors were chosen [10]. An inappropriate selection can lead to a misalignment of expectations in supervisee-supervisor relationships affecting further doctoral experience [11].

Few studies have focused on the factors influencing this choice. The choice may be based on rational calculations or the expected behaviors of the supervisor, but it may also rest on irrational foundations [9]. Researchers typically emphasize a supervisor's academic attributes, reputation, overall supervision experience, and demographic factors as the most frequent criteria for the selection of a supervisor [6,9,12]. However, most of these studies have identified

the factors affecting selection but have not assessed the relationship between these factors and students' satisfaction with the interaction process, as well as students' consequent results. Our study addresses this gap by examining how various selection strategies contribute to the difficulties during doctoral training, and students' confidence that they will successfully defend a dissertation. Using data from a nationwide survey of Russian doctoral students conducted in 2022, we aim to answer the following research questions:

> *RQ1: Are different supervisor selection strategies related to students' consequent difficulties in interaction with a supervisor?*
>
> *RQ2: Are different supervisor selection strategies related to student's confidence in a successful future defense?*

Navigating the selection strategies: a literature review

The relationships between doctoral students and their supervisors play a crucial role in the quality of the doctoral experience, the completion of a dissertation, and the overall well-being of doctoral students [4,5,13]. Research often focuses on the processes during doctoral training, but less attention is paid to the initial stages of student-supervisor relationship formation and development. However, studies have shown that the compatibility between doctoral students and their supervisors, including academic interests and working styles, is significantly related to students' satisfaction and success [14,15]. Although in some countries and universities supervisors are appointed by the administration, researchers note that the selection process is often informal, allowing doctoral students to influence it [15–17]. Thus the "right" choice of the supervisor is crucial for matching the expectations of a doctoral student and a supervisor regarding work styles and necessary support [18].

In this part, we review the criteria for choosing a supervisor highlighted in previous research, starting from the most common ones, related to academic attributes, and finishing by less frequently examined criteria such as demographic characteristics and pragmatic benefits.

One of the most explored criteria in the selection of a supervisor is based on her or his academic attributes and expertise [10]. The importance of a supervisor's academic achievements and qualifications is particularly relevant to the specific topic of the dissertation, rather than in a general sense [12,19–21]. Research has shown that such choices lead to satisfaction when working with a supervisor, especially if supervisors are highly experienced in the research topic [6]. Doctoral students evaluate a supervisor's academic experience based on the quantity of publications, research grants, and educational background [22,23].

Relevant research has demonstrated that doctoral students are also guided by previous interactions with their supervisors [9,24]. Established relationships built earlier become an important factor in the selection process. Typically, a positive relationship with a supervisor can enhance student experience and increase research and educational results [25,26]. However, qualitative studies have investigated that this factor can be negative if a supervisor fails to provide adequate academic support [15,24]. Some studies have examined the negative effects associated with mixing of academic and non-academic interests, e.g., in case of romantic relationships between supervisees and supervisors [27].

Supervisor's previous supervision experience also stands out as a significant choice factor [22]. Studies have highlighted the value of a supervisor's background in supervision as a positive factor for defending a dissertation [15]. This connection may likely be explained in such a way that experienced supervisors have a clearer understanding of the doctoral research process and requirements and a proven ability to guide students through complex academic, sometimes bureaucratic, procedures. Students may evaluate the supervision experience of faculty based

on recommendations and reputation among previous students, colleagues, and a department [12,28].

Some studies investigated the effects of demographic characteristics of supervisors such as age, gender, race, and nationality. These papers have shown the significance of a supervisor's gender and age in student success, and the effects of these factors can vary [29]. However, it is quite rare that the choice of a supervisor is fully determined by these factors [10,30]. Less frequently mentioned criteria relate to pragmatic benefits such as the availability of the supervisor in terms of time, research funding, financial status, and administrative position at the university [22,30,31].

Although there is a plethora of research dedicated to the selection criteria and strategies, few empirical quantitative studies investigate their consequences and effects. Even the relevant papers reviewed in this section have some methodological limitations related to the local nature of surveys (surveys are limited to one particular university) and small sample sizes, which does not allow for generalizing the obtained findings. Our study aims to address these shortcomings by analyzing data from a nationwide survey of Russian doctoral students.

Doctoral supervision in the context of Russian doctoral education

Doctoral education in Russia is provided by several types of organizations: universities (87% of the doctoral student body), research institutes (12%), and other organizations (less than 1%). According to the most recent data from the Federal State Statistics Service (2024), 45,075 students were enrolled in doctoral programs in 2022, while only 1,791 doctoral degrees were awarded in the same year [32]. More than half of Russian doctoral students are male (66%) and have state-funded positions (53%).

Doctoral education in Russia has been experiencing challenges and transformations during the past 10 years [33]. Alarming tendencies are related to the declining number of students, and graduates, as well as completion and defense rates. Although the reasons for the crisis of Russian doctoral education are complex, poor supervision is considered one of the key factors contributing to the current “crisis of Russian doctoral education” [33,34]. Despite the reform of transition from the traditional master-apprenticeship model to the structured model of doctoral programs, which took place in 2012-2013, supervisors in Russia are still the main figures in a doctoral journey which produces the problem of students’ excessive dependence on them [35]. At the same time, various empirical studies show that approximately 16% of supervisors in Russia can be described as “hands-off supervisors”, i.e., those who do not perform any functions associated with the supervisor role [36], practices of team supervision are rare [37] and not regulated normatively [38], and enhancement of the supervision quality is the least frequent measure mentioned in the development programs of Russian leading universities [39]. Academics explain poor quality of supervision by a combination of factors including supervisors’ time limitations and high workload for other activities such as research and teaching, a lack of professional development programs, and insufficient incentives for supervisors [40]. These circumstances are only worsened by high academic requirements imposed on doctoral students (he or she should have at least 3 papers in highly-ranked journals), as well as a high level of bureaucracy associated with the final stages of doctoral training when the help of the supervisor is also necessary (e.g., to find external reviewers, organize the pre-defense and defense, prepare the review on a thesis). Some universities are provided with the right to award their degrees and can simplify these procedures, however, their requirements regarding students’ publications cannot be lower than the national ones.

Criteria and procedures for supervisor appointment in Russia are regulated at the national, institutional, and departmental levels [40]. National-level regulations provide minimal

requirements to the qualification and research productivity of a supervisor: he or she has to have a research degree and conduct research on the thesis topic of a doctoral student in recent years, as well as have publications on this specialization in the previous three years. Additionally, it sets a deadline for the official supervisor appointment, which is now 30 days after students' enrolment. Institutional- and departmental-level regulations serve as complementary rules to the national ones. To work as a supervisor, a candidate who meets the criteria should be approved by the corresponding department at the university. Russian doctoral students have a right to choose a supervisor, or the latter may be suggested by the department. While it is hard to empirically estimate the prevalence and diversity of practices at the stage of the supervisor selection, research at least shows that only 19% of Russian universities require doctoral candidates to have preliminary agreements with their potential supervisor at the admission stage [41] indicating that the process of appointment usually takes place after the student was enrolled in a program.

Data

In this study, we used data from a nationwide survey carried out under the framework of the "Monitoring of Education Markets and Organizations" (MEMO) project. The project was supported by the Russian Ministry of Science and Higher Education (RMSHE). Data were collected from May 31 to August 31, 2022. The target population of the survey was students who studied in doctoral programs in Russian universities and research institutes in the 2021-2022 academic year.

Universities and research institutes were invited by the RMSHE to participate in the study and were requested to distribute a link to the questionnaire among their doctoral students. The survey was arranged online, participation was voluntary. The data were obtained in an anonymized form to ensure that participants could not be identified during or after the data

collection. Informed consent was obtained at the beginning of the online survey. At the first page of the survey, respondents were required to check a box indicating their agreement to participate in the study. The study was reviewed and approved by the Institutional Review Board at HSE University.

A total of 2392 doctoral students completed the questionnaire. For our study, we focused on those enrolled at universities, excluding students from research institutes, who represented only 15% of our sample and 10-13% of all doctoral students in Russia. Their specific characteristics and experiences were not the primary focus of our investigation. Additionally, fifth-year students, as well as students specializing in medicine and agriculture, were excluded from our analysis due to their small representation in the sample. In this regard, the generalization of the study results to the excluded groups is not relevant.

Ultimately, 1796 participants were included in our analysis. Among them, 40% studied at leading universities, i.e. universities that participate in the academic excellence program “Priority 2030” or hold at least one of the following special statuses: national research university, federal university, or member of the Association of the Leading Universities. Among other things, the presence of such statuses implies that universities introduce various measures to enhance the visibility, attractiveness, and performance of their doctoral programs. Over half (53%) of the sample were female students. Nearly two-thirds of the participants were first- (36%) and second-year (33%) students. The majority (83%) were full-time students, and 71% had a tuition-free form of financing. In terms of the major fields of study, 25% were from the social sciences, 23% from technology, 23% from natural sciences and mathematics, 17% from the humanities, and 12% from educational sciences.

Materials and methods

The survey comprised 11 thematic blocks addressing the period before and during the doctoral studies, as well as expectations about the future. It also collected socio-demographic characteristics.

To identify various supervisor selection strategies, we used the following multiple-choice question:

Which of the following did you use as criteria when choosing a supervisor?

1. *The authority and expertise of the supervisor on your dissertation topic*
2. *Supervisor's number of publications and/or presentations at scientific conferences*
3. *The alignment of the supervisor's research interests with yours*
4. *The supervisor has doctoral students who successfully defended their dissertations*
5. *Good feedback about the supervisor from other doctoral students or faculty*
6. *Successful previous experience of interaction with the supervisor*
7. *Friendship with the supervisor*
8. *There were no other faculty members who worked on your research topic*

The most prevalent options chosen by participants are related to the authority and expertise of the supervisor (64%) and aligned research interests (59%). The least frequent option is a lack of other supervisors on the topic of interest (11%). See the detailed distribution in Table 1.

Table 1. Prevalence of answers to the question: "Which of the following did you use as criteria when choosing a supervisor?" (N=1796)

Option	Prevalence
The authority and expertise of the supervisor on your dissertation topic	64%
The alignment of the supervisor's research interests with yours	59%

Option	Prevalence
Good feedback about the supervisor from other doctoral students or faculty	40%
Successful previous experience of interaction with the supervisor	40%
The supervisor has doctoral students who successfully defended their dissertations	32%
Supervisor’s number of publications and/or presentations at scientific conferences	25%
Friendship with the supervisor	25%
There were no other faculty members who worked on your research topic	11%

To be used in the consequent analysis, presented options were merged into several components, which are referred to as selection strategies, using principal component analysis (PCA). We employed PCA on the tetrachoric correlation matrix since variables were presented as binary ones. Promax rotation was used because we expected different selection strategies to be correlated. Analysis was conducted in *R* using the *psych* package (Revelle, 2022). To determine the optimal number of components, we compared models with 2 to 5 components. Based on explained variance and interpretability, we selected the model with four components. The selected model explained 74% of the variance. The extracted components represent the following reasons that can be used to select a supervisor: 1) subject-matter expertise, 2) successful supervision history, 3) positive personal relationships, and 4) the “only option”. Factor loadings and communalities for the final model are presented in Table 2.

Table 2. Factor loadings and communalities of the PCA model

	RC1	RC2	RC3	RC4	Communalities
The authority and expertise of the supervisor on your dissertation topic	0.701	0.072	-0.152	-0.247	0.58
Supervisor’s number of publications and/or presentations at scientific conferences	0.688	0.351	-0.035	0.246	0.66

	RC1	RC2	RC3	RC4	Communalities
The alignment of the supervisor's research interests with yours	0.825	-0.265	0.171	-0.003	0.78
The supervisor has doctoral students who successfully defended their dissertations	-0.008	0.839	-0.031	-0.085	0.71
Good feedback about the supervisor from other doctoral students or faculty	-0.064	0.855	0.114	-0.020	0.75
Successful previous experience of interaction with the supervisor	0.065	0.000	0.820	-0.129	0.69
Friendship with the supervisor	-0.025	0.081	0.868	0.087	0.77
There were no other faculty members who worked on your research topic	-0.015	-0.065	-0.023	0.950	0.91

Note. RC1 stands for subject-matter expertise, RC2 is successful supervision history, RC3 is positive personal relationships, and RC4 corresponds to the "only option".

We then calculated component scores for each participant using the *thurstone* method. These scores are standardized continuous variables and can be interpreted as to what extent each participant relied on various groups of criteria when selecting a supervisor. Higher scores indicate a stronger alignment with a particular group of criteria.

The results of statistical tests for the extracted components are presented in Table 3. The "only option" strategy shows significant gender-based differences being less relevant for females. Successful supervision history is more common when doctoral students choose male supervisors. Students who study tuition-free are more likely to choose their supervisors based on subject-matter expertise and positive personal relationships. In contrast, tuition-based doctoral students prioritize the successful supervision history of potential supervisors. Part-time students less frequently rely on factors related to subject-matter expertise and successful supervision history, and also less frequently face the "only option" setting. At leading

universities, students more frequently choose supervisors based on subject-matter expertise and encounter the “only option” scenario, whereas successful supervision history is less common among students of these organizations. The selection of supervisors based on subject-matter expertise, positive personal relationships, and the “only option” scenario is more common when the supervisor is younger. Conversely, older supervisors are more frequently chosen based on their successful supervision history. Doctoral students in STEM fields, i.e., engineering and technology, mathematics and natural sciences, prioritize positive personal relationships and successful supervision history.

Table 3 Differences in the average values of components

Variable	Category	Subject-matter expertise	Positive personal relationships	Successful supervision history	Only option
Gender of a doctoral student	**Female**	-0.01	-0.04	0.03	-0.04
	**Male**	0.02	0.04	-0.03	0.05
	**T-statistic**	-0.57	-1.47	1.16	-1.97*
Gender of a supervisor	**Female**	0.03	-0.03	-0.08	-0.05
	**Male**	-0.02	0.02	0.06	0.04
	**T-statistic**	-0.81	1.07	2.51*	1.85
Form of financing	**Tuition-free**	0.06	0.1	-0.04	0.02
	**Tuition-based**	-0.16	-0.3	0.1	-0.06
	**T-statistic**	3.48***	7.04***	-2.32*	1.45
Form of study	**Full-time**	0.04	0.06	-0.0	0.03
	**Part-time**	-0.2	-0.3	0.01	-0.14
	**T-statistic**	3.22**	5.14***	-0.24	2.6**
University status	**Not leading university**	-0.08	-0.02	0.15	-0.06
	**Leading**	0.12	0.02	-0.22	0.08

Variable	Category	Subject-matter expertise	Positive personal relationships	Successful supervision history	Only option
	**university**				
	**T-statistic**	-3.74***	-0.78	7.01***	-2.84**
Age of a supervisor	**60 or more**	-0.08	-0.11	0.17	-0.13
	**50-59**	-0.06	-0.07	0.06	0.01
	**40-49**	0.12	0.1	-0.13	0.02
	**39 or less**	0.09	0.36	-0.38	0.22
	**F-statistic**	3.27*	11.26***	14.54***	6.08***
Field of study	**Engineering and technology**	-0.09	0.14	0.23	0.02
	**Humanities**	0.1	0.06	-0.12	0.01
	**Pedagogy and education**	-0.13	-0.28	-0.0	-0.11
	**Social science**	0.04	-0.14	-0.16	-0.03
	**Mathematics and natural science**	0.04	0.11	0.03	0.06
	**F-statistic**	2.1	8.83***	7.49***	1.08

We propose two sets of models to examine the relationship between selection strategies and various outcomes. Each set contains several models, into which we sequentially incorporated predictors and control variables.

The first set consists of models where a dependent variable describes whether a student reports difficulties in interaction with a supervisor during their doctoral studies. This set of models was built using binary logistic regression. We used a general survey question “To what extent have the following factors hindered your progress in the doctoral program?”, which, among other

options, included the “difficulties in interacting with the supervisor” option. In our sample, 22% of the participants reported difficulties in interacting with the supervisor (N=1606, excluding those who found it difficult to evaluate).

The second set comprises linear regression models. As a dependent variable, we used a standardized variable reflecting students’ lack of confidence about completing their dissertation, which was proposed in previous research [37] and combined students’ agreement with the following statements (measured on a scale from 1 to 4):

“I am afraid…”

1. *I will not defend my dissertation in time*
2. *I will not pass my next examination*
3. *All my work is being done in vain and does not get me closer to the defense*
4. *That my research does not correspond to the level of a doctoral dissertation*
5. *I am not capable of completing my dissertation*

Extraction of the component was carried out using categorical principal component analysis (CatPCA; in *R* using the *princals* class of the *Gifi* package [42]). The resulting model with one component explained 70% of the variance, component loadings and communalities for this model are presented in Table 4. The resulting variable was a standardized continuous one with a higher value indicating students’ lower confidence in the defense (N=1497, excluding missing data).

Table 4. Factor loadings and communalities for the CatPCA model

I am afraid…	**Loadings**	**Communalities**
I will not defend my dissertation in time	0.809	0.655
I will not pass my next examination	0.772	0.596

I am afraid…	Loadings	Communalities
All my work is being done in vain and does not get me closer to the defense	0.872	0.760
That my research does not correspond to the level of a doctoral dissertation	0.838	0.702
I am not capable of completing my dissertation	0.883	0.780

Into both sets of models, we also incorporated a set of control variables commonly associated with doctoral students' experience and success.

In the first set of models, we included control variables that could be related to satisfaction with supervisor-supervisee interaction. The first variable indicates whether a student finds the frequency of meetings with a supervisor sufficient, which may be one of the indicators of matching working styles. For analytical purposes, we recoded this variable from ordinal into a binary format ("Fully sufficient" and "Rather sufficient" coded as 1; "Rather inadequate" and "Quite inadequate" coded as 0). Previous studies have highlighted the significance of this predictor and its impact on doctoral students' experience [43]. The second control variable is the age of the supervisor, treated as an ordinal variable. This characteristic is directly related to the supervisor's scientific authority, supervision experience, and position at the university. It may be associated with different styles of supervision and requirements expected from students.

For both sets of models, we considered the presence and type of students' employment during their doctoral training as another control variable. A significant proportion of doctoral students in Russia (90%) are employed during their studies [44]. The effects of employment on doctoral outcomes are heterogenous. For example, on-campus employment related to research duties during doctoral training can increase the likelihood of dissertation defense. In contrast, off-campus employment and non-research jobs at the university are negatively associated with the completion of a dissertation [45]. In terms of satisfaction with supervision, employed doctoral

students are usually considered nontraditional ones (see more on the term: [46]), and such students are characterized by more autonomous style of work and higher levels of satisfaction with their overall doctoral experience [47]. Drawing on the design of previous studies [45,48], we categorized employment status into four types to control possible heterogeneous effects: unemployed (10%), employed outside the university (50%), employed in a research position within the university (14%), and employed in non-research positions within the university (26%). This variable was constructed from responses to several survey questions: “Are you currently employed?”, “Do you work at the same university where you are currently studying?”, and “What type of position do you hold?” (options included “research position”, “teaching position”, “administrative position”, and “other”).

Additionally, all our models included formal control variables such as the year of study, form of study, form of financing, type of university (leading or not), field of study, and gender.

Missing data were handled using listwise-deletion strategy separately for each model.

Results

Difficulties in interaction with supervisor

The first set of models predicts whether the student faces difficulties in interaction with their supervisor. Results of the corresponding logistic regression models are presented in Table 5 (odds ratios and standard errors).

Table 5. Results of binary logistic regression (DV — facing difficulties in interaction with a supervisor, yes/no)

Variable	Model 1		Model 2		Model 3		Model 4	
	Exp(B)	SE	Exp(B)	SE	Exp(B)	SE	Exp(B)	SE
Intercept	0.165***	0.293	0.853	0.352	1.034	0.363	1.231	0.42

Variable	Model 1		Model 2		Model 3		Model 4	
	Exp(B)	SE	Exp(B)	SE	Exp(B)	SE	Exp(B)	SE
Subject-matter expertise	0.607***	0.061	0.669***	0.069	0.67***	0.069	0.657***	0.07
Positive personal relationships	0.649***	0.067	0.65***	0.074	0.664***	0.075	0.653***	0.076
Successful supervision history	0.84**	0.064	0.857*	0.071	0.838*	0.072	0.839*	0.072
Only option	1.625***	0.056	1.487***	0.064	1.512***	0.065	1.499***	0.065
Satisfied with the frequency of meetings			0.107***	0.156	0.104***	0.157	0.104***	0.158
Age of supervisor (ref = "60 and more")								
39 and less					0.636	0.263	0.616	0.264
40-49					0.68*	0.191	0.673*	0.192
50-59					0.75	0.186	0.751	0.187
Employment status (ref = "not employed")								
Employed in a research position							1.158	0.289
Employed in other position							0.757	0.268
Employed outside the university							0.717	0.242
1st year	0.812	0.227	0.687	0.251	0.703	0.252	0.748	0.255
2nd year	0.757	0.228	0.704	0.253	0.696	0.253	0.734	0.255
3rd year	0.984	0.236	0.863	0.263	0.863	0.263	0.88	0.265
Full-time	1.416	0.267	1.901*	0.308	1.974*	0.31	1.883*	0.314
Tuition-free	1.157	0.231	1.108	0.258	1.104	0.259	1.105	0.262
Female	1.115	0.137	1.07	0.154	1.077	0.154	1.079	0.156

Variable	Model 1		Model 2		Model 3		Model 4	
	Exp(B)	SE	Exp(B)	SE	Exp(B)	SE	Exp(B)	SE
Field of study (ref="Mathematics and natural science")								
Humanities	1.035	0.213	0.871	0.241	0.877	0.242	0.941	0.247
Education and pedagogy	0.942	0.239	0.922	0.264	0.948	0.266	1.017	0.27
Social science	0.803	0.204	0.698	0.229	0.724	0.231	0.768	0.233
Engineering and technology	1.243	0.185	1.142	0.207	1.126	0.207	1.202	0.211
Leading university	1.283	0.143	1.169	0.16	1.176	0.161	1.137	0.162
Observations	1606		1566		1566		1566	
R^2 McFadden	0.105		0.220		0.240		0.244	

*Note: * p < 0.05, ** p < 0.01, *** p < 0.001*

Model 1 demonstrates that selecting supervisors based on their subject-matter expertise, as well as positive personal relationships, significantly reduces the odds of experiencing interaction difficulties during doctoral training. Specifically, a one standardized unit increase in the component related to the subject-matter expertise reduces the odds by 39%, while prioritizing positive personal relationships decreases the odds by 35% per unit. Additionally, having a supervisor with a successful supervision history also markedly decreases interaction difficulties. In contrast, selecting a supervisor out of necessity, when no other options are available, increases the odds of consequent interaction difficulties by 63% per one standardized unit.

In Models 2, 3, and 4, we included control variables concerning satisfaction with the frequency of meetings with a supervisor, the age of the supervisor, and the student’s employment status.

Despite the significant coefficients of some variables, the effects of the analyzed selection strategies remained significant, exhibiting similar effects as those observed in the first model.

Lack of confidence about defense

The second set of models predicts the level of students' uncertainty regarding the future defense. The results of the corresponding linear regression models are presented in Table 6.

Table 6. Results of linear regression analysis (DV — lack of confidence about future successful defense)

Variable	Model 1				Model 2			
	Estimate	SE	LB CI 95%	UB CI 95%	Estimate	SE	LB CI 95%	UB CI 95%
Intercept	-0.4***	0.1	-0.6	-0.19	-0.32*	0.14	-0.58	-0.05
Subject-matter expertise	-0.18***	0.02	-0.22	-0.14	-0.18***	0.02	-0.22	-0.13
Positive personal relationships	-0.04	0.02	-0.08	0.01	-0.03	0.02	-0.08	0.01
Successful supervision history	-0.06**	0.02	-0.1	-0.02	-0.07**	0.02	-0.12	-0.02
Only option	0.14***	0.02	0.1	0.19	0.15***	0.03	0.1	0.2
1st year	0.01	0.09	-0.16	0.17	-0.09	0.09	-0.27	0.09
2nd year	0.13	0.09	-0.04	0.3	0.08	0.09	-0.1	0.26
3rd year	0.12	0.09	-0.05	0.3	0.12	0.1	-0.07	0.31
Employment status ref="not employed"								
Employed in a research position					-0.33**	0.1	-0.53	-0.12
Employed in other position					-0.04	0.09	-0.22	0.15
Employed					-0.05	0.09	-0.22	0.12

Variable	Model 1				Model 2			
	Estimate	SE	LB CI 95%	UB CI 95%	Estimate	SE	LB CI 95%	UB CI 95%
outside the university								
Full-time	0.07	0.09	-0.11	0.24	0.11	0.1	-0.08	0.31
Tuition-free	0.05	0.08	-0.11	0.2	0.08	0.09	-0.09	0.25
Female	0.17***	0.05	0.07	0.26	0.17**	0.05	0.06	0.27
Field of study ref="Mathematics and natural science"								
Humanities	0.18*	0.08	0.03	0.33	0.11	0.09	-0.06	0.27
Education and pedagogy	0	0.09	-0.17	0.17	-0.05	0.09	-0.23	0.14
Social science	0.1	0.07	-0.04	0.24	0.12	0.08	-0.03	0.27
Engineering and technology	0.11	0.07	-0.02	0.24	0.08	0.07	-0.06	0.23
Leading University	0.16**	0.05	0.05	0.26	0.19***	0.06	0.08	0.3
Observations	1497				1497			
Adj R^2	0.075				0.082			

Note: * $p < 0.05$, ** $p < 0.01$, *** $p < 0.001$

The first linear model demonstrates the significance of selection strategies based on subject-matter expertise, successful supervision history, and the "only option" factor. The first two factors are associated with a decrease in lack of confidence, with subject-matter expertise showing the strongest negative association; specifically, this strategy reduces the dependent variable by 0.18 per standardized unit, all other things being equal. Similarly, choice based on successful supervision history reduces the lack of confidence by 0.06 per standardized unit. However, selecting a supervisor as the "only option" is positively associated with a lack of confidence. Notably, reliance on positive personal relationships when choosing a supervisor

does not significantly predict outcomes in this model, contrasting with previous logistic models predicting difficulties in interaction with a supervisor.

In the second model, we included employment status as a control variable. Working in a research position at their university is shown to decrease students' lack of confidence, consistent with findings from previous research [45,48]. The effects of the predictors related to supervisor selection strategies in Model 2 mirror those in the first model.

Discussion and Conclusion

The purpose of the current study was to determine the relationship between various strategies doctoral students employ when selecting their supervisors and two key outcome variables: whether students face any difficulties in interaction with supervisors during their consequent training and how confident they are in future successful dissertation defense. Based on the analysis conducted, we draw several important findings.

First, we demonstrated that selection based on such an academic criterion as the supervisor's academic expertise is the most effective among other possible strategies. Choosing supervisors based on their expertise in the dissertation topic, doctoral students reduce the likelihood of encountering problems with them during the training period, as well as feel more confident regarding defending a dissertation. Additionally, positive personal relationships and successful supervision history have also shown significant associations in reducing the probability of facing difficulties with the supervisor. These results generally align with previous findings which emphasized the significance of academic attributes and work-oriented style of relationships [6,10,12,22].

Second, we discovered that although selection based on positive personal relationships reduces the chances of facing difficulties with supervisors, we found no significant association between this component and students' levels of confidence about future defense. We interpret this

finding as confirming the results of qualitative studies about bidirectional and complex effects of this criterion: positive relationships alone, without additional support such as strong academic expertise or successful supervision background, do not necessarily help students in navigating their doctoral challenges [15,24].

Third, the only strategy that provides negative results both regarding facing difficulties with supervisor and students' levels of confidence is related to the "only option" scenario, i.e., the situation when students have no other choice but to work with a particular supervisor specializing in a student topic. As subject-matter expertise is controlled in all models, the negative coefficients are reached by the very fact of the absence of alternatives, which aligns with the results of previous studies [15]. This scenario and the revealed negative effects can be interpreted in several ways. On the one hand, this scenario may indicate the general deficit of potential supervisors that, considering the high requirements imposed on them [40] and declining number of scientific degree holders in Russia, may indeed be relevant to some Russian universities. In this case, one of the potential practical solutions may be related to the introduction of team or co-supervision practices that become the standard in some national contexts [49], but are still scarce in Russia [37]. While one of the supervisors may be in charge of the subject expertise and meet all the formal criteria, the second one may be chosen among younger scholars who may provide methodological, organizational, or informational support, thus receiving the experience necessary for future supervision practice. On the other hand, this scenario may be also explained by students' unawareness of the specialists within the university. In this case, universities and departments should provide more detailed information about the available supervisors and their topics of expertise, as well as their supervision history, and encourage students to make informed choices rather than constrained ones.

When interpreting the results, one needs to take into account the limitations associated with the conducted research. First of all, we focus only on the criteria students use when selecting their

final supervisor but lack detailed information about the circumstances in which this selection was made. As it was mentioned in the national context section, it is not always the case that students themselves choose their supervisors, the latter can be suggested and/or appointed by the department as well, but we cannot differentiate these alternatives based on our data. Thus, our findings are more relevant for national and institutional contexts that assume that students themselves choose their supervisors. Second, we do not know much about nature, diversity, and the extent of difficulties that students face when interacting with their supervisors (our first dependent variable). It is plausible that distinct selection strategies may be linked to diverse issues in interaction, and further investigation is required to delve deeper into these variations. Third, as we rely on cross-sectional data, we do not make any conclusions about causal relationships between our variables, as well as we do not know how various selection strategies are related to the actual performance of doctoral students (because we do not have such kinds of variables). Finally, we could encounter a survival bias in our research as our survey does not cover those students who, for any reason, decided to drop out during their studying, particularly because of some kind of supervisor-supervisee mismatch or other issues related to interaction with a supervisor. This methodological constraint may lead to over- or under-estimation of some of the identified effects.

Funding

Both DP and SZ received support from the project "International academic cooperation" at HSE University. The funder had no role in study design, data collection and analysis, decision to publish, or manuscript preparation.

Acknowledgements

The authors would like to thank Evgeniy Terentev, Nikita Smirnov, and Jennet Babaeva for their valuable comments on the initial version of the manuscript.

References

1. Bowen WG, Rudenstine NL. In pursuit of the PhD. Princeton, N.J.: Princeton University Press; 1992. p. 60
2. McAlpine L, McKinnon M. Supervision – the most variable of variables: student perspectives. Stud Contin Educ. 2013;35: 265–280. doi:10.1080/0158037X.2012.746227
3. Gisemba Bagaka's J, Bransteter I, Rispinto S, Badillo N. Exploring Student Success in a Doctoral Program: The Power of Mentorship and Research Engagement. Int J Dr Stud. 2015;10: 323–342. doi:10.28945/2291
4. Sverdlik A, C. Hall N, McAlpine L, Hubbard K. The PhD Experience: A Review of the Factors Influencing Doctoral Students' Completion, Achievement, and Well-Being. Int J Dr Stud. 2018;13: 361–388. doi:10.28945/4113
5. Mainhard T, Van Der Rijst R, Van Tartwijk J, Wubbels T. A model for the supervisor–doctoral student relationship. High Educ. 2009;58: 359–373. doi:10.1007/s10734-009-9199-8
6. Zhao C, Golde CM, McCormick AC. More than a signature: how advisor choice and advisor behaviour affect doctoral student satisfaction. J Furth High Educ. 2007;31: 263–281. doi:10.1080/03098770701424983
7. Bao Y, Kehm BM, Ma Y. From product to process. The reform of doctoral education in Europe and China. Stud High Educ. 2018;43: 524–541. doi:10.1080/03075079.2016.1182481
8. Salinas-Perez JA, Rodero-Cosano ML, Rigabert A, Motrico E. Actions and techniques in supervision, mentorships and tutorial activities to foster doctoral study success: A scoping literature review. Int J Educ Res. 2019;96: 21–31. doi:10.1016/j.ijer.2019.05.004

9. Shafiq A, Jan A. Factors Influencing Gen-Y Undergraduates' Choice of Research Supervisor: A Case Study of a Malaysian Private University. Educ Process Int J. 2017;6: 20–34. doi:10.22521/edupij.2017.64.2
10. Shafiq A, Pahlevan Sharif S, Jan A. Psychometric Analysis of a Proposed Model to Determine Factors Influencing Selection of a Research Supervisor. Int J Dr Stud. 2020;15: 285–304. doi:10.28945/4567
11. Moxham L, Dwyer T, Reid-Searl K. Articulating expectations for PhD candidature upon commencement: ensuring supervisor/student 'best fit.' J High Educ Policy Manag. 2013;35: 345–354. doi:10.1080/1360080X.2013.812030
12. Onen D. Factors influencing student choice of the research supervisor: A study of doctoral students. South Africa: South Africa International Conference on Education; 2016. Available: https://nru.uncst.go.ug/server/api/core/bitstreams/447dbaa2-d441-4d57-b707-437eb68c3374/content
13. Devos C, Boudrenghien G, Van Der Linden N, Azzi A, Frenay M, Galand B, et al. Doctoral students' experiences leading to completion or attrition: a matter of sense, progress and distress. Eur J Psychol Educ. 2017;32: 61–77. doi:10.1007/s10212-016-0290-0
14. Dericks G, Thompson E, Roberts M, Phua F. Determinants of PhD student satisfaction: the roles of supervisor, department, and peer qualities. Assess Eval High Educ. 2019;44: 1053–1068. doi:10.1080/02602938.2019.1570484
15. Ives G, Rowley G. Supervisor selection or allocation and continuity of supervision: Ph.D. students' progress and outcomes. Stud High Educ. 2005;30: 535–555. doi:10.1080/03075070500249161
16. Phillips EM, Pugh DS. How to get a PhD: a handbook for students and their supervisors. 5. ed., repr. Maidenhead: Open Univ. Press; 2012.

17. Donald JG, Saroyan A, Denison DB. Graduate student supervision policies and procedures: A case study of issues and factors affecting graduate study. Can J High Educ. 1995;25: 71–92. doi:10.47678/cjhe.v25i3.183223
18. Van Rooij E, Fokkens-Bruinsma M, Jansen E. Factors that influence PhD candidates' success: the importance of PhD project characteristics. Stud Contin Educ. 2021;43: 48–67. doi:10.1080/0158037X.2019.1652158
19. MacDougall M. Predicting supervisor capacities to foster higher forms of learning through undergraduate medical student research. J Scholarsh Teach Learn. 2014; 75–94. doi:10.14434/josotl.v14i1.3245
20. Momeni M, Samimi B, Afshari MA, Maleki MH, Mohammadi J. Selection Process of Supervisor for Doctoral Dissertation Using Analytical Network Process (ANP): An Iranian Study. J Manag Strategy. 2011;2: p63. doi:10.5430/jms.v2n2p63
21. Ray S. Selecting a Doctoral Dissertation Supervisor: Analytical Hierarchy Approach to the Multiple Criteria Problem. Int J Dr Stud. 2007;2: 023–032. doi:10.28945/55
22. Datta S, Beriha G, Patnaik B, Mahapatra S. Use of compromise ranking method for supervisor selection: A multi-criteria decision making (MCDM) approach. Int J Vocat Tech Educ. 2009;1: 7–13.
23. Buttery EA, Richter EM, Leal Filho W. An overview of the elements that influence efficiency in postgraduate supervisory practice arrangements. Int J Educ Manag. 2005;19: 7–26. doi:10.1108/09513540510574920
24. Manderson D. Asking Better Questions: Approaching the Process of Thesis Supervision. J Leg Educ. 1996;46: 407–419.
25. Pyhältö K, Vekkaila J, Keskinen J. Fit matters in the supervisory relationship: doctoral students and supervisors perceptions about the supervisory activities. Innov Educ Teach Int. 2015;52: 4–16. doi:10.1080/14703297.2014.981836

26. Kolmos A, Kofoed LB, Du XY. PhD students’ work conditions and study environment in university- and industry-based PhD programmes. Eur J Eng Educ. 2008;33: 539–550. doi:10.1080/03043790802588383
27. Goodyear RK, Crego CA, Johnston MW. Ethical issues in the supervision of student research: A study of critical incidents. Prof Psychol Res Pract. 1992;23: 203–210. doi:10.1037/0735-7028.23.3.203
28. Grant B. Walking on a rackety bridge: mapping supervision. Melbourne; 1999. Available: https://www.researchgate.net/profile/Grant-Barbara-2/publication/228505875_Walking_on_a_rackety_bridge_mapping_supervision/links/56c4287a08ae602342513696/Walking-on-a-rackety-bridge-mapping-supervision.pdf
29. Ditkovsky K, Fridlyanova S, Gokhberg L, Kotsemir M, Kuznetsova I, Martynova S, et al. Science and Technology Indicators in the Russian Federation: Data book. Gokhberg L, Kuzminov Y, editors. Moscow: National Research University Higher School of Economics; 2022. Available: https://publications.hse.ru/pubs/share/direct/779594026.pdf
30. Jamieson S, Gray C. The Supervision of Undergraduate Research Students: Expectations of Student and Supervisor. Pract Evid Scholarsh Teach Learn High Educ. 2006;1: 37–59.
31. McCallin A, Nayar S. Postgraduate research supervision: a critical review of current practice. Teach High Educ. 2012;17: 63–74. doi:10.1080/13562517.2011.590979
32. Federal State Statistics Service. The main indicators of the doctoral training in the Russian Federation. 2024. Available: https://rosstat.gov.ru/statistics/education
33. Maloshonok N, Terentev E. National barriers to the completion of doctoral programs at Russian universities. High Educ. 2019;77: 195–211. doi:10.1007/s10734-018-0267-9

34. Terentiev EA, Bekova SK, Maloshonok NG. The crisis of postgraduate studies in russia: what bears problems and how to overcome them. Univ Manag Pract Anal. 2018;22: 54–66. doi:10.15826/umpa.2018.05.049
35. Maloshonok N, Terentev E. Towards the New Model of Doctoral Education: The Experience of Enhancing Doctoral Programs in Russian Universities. Vopr Obraz Educ Stud Mosc. 2019; 8–42. doi:10.17323/1814-9545-2019-3-8-42
36. Gruzdev I, Terentev E, Dzhafarova Z. Superhero or hands-off supervisor? An empirical categorization of PhD supervision styles and student satisfaction in Russian universities. High Educ. 2020;79: 773–789. doi:10.1007/s10734-019-00437-w
37. Zhuchkova S, Terentev E, Saniyazova A, Bekova S. Departmental academic support for doctoral students in Russia: Categorisation and effects. High Educ Q. 2023;77: 215–231. doi:10.1111/hequ.12389
38. Terentev E, Kuznetsov P. Russia. In: Kumar V, Wald N, editors. Global Perspectives on Enhancing Doctoral Co-Supervision. Singapore: Springer Nature Singapore; 2024. pp. 75–86. doi:10.1007/978-981-97-0460-6_8
39. Zhuchkova SV, Pavliuk DM. Is Doctoral Education A Priority? Doctoral Education Improvement as an Element of “Priority-2030” Universities’ Development Programs. Univ Manag Pract Anal. 2024;28: 21–33. doi:10.15826/umpa.2024.01.002
40. Bekova S, Maloshonok N, Terentev E. Russia. First Edition. In: Taylor S, Kiley M, Holley KA, editors. The making of doctoral supervisors: international case studies of practice. First Edition. London: Routledge; 2020.
41. Zhuchkova SV. How is Doctoral Students’ Admission Arranged at Russian Universities? Univ Manag Pract Anal. 2022;26: 92–104. doi:10.15826/umpa.2022.02.015
42. Mair P. Gifi Methods. Modern Psychometrics with R. Cham: Springer International

Publishing; 2018. pp. 231–256. doi:10.1007/978-3-319-93177-7_8

43. Seeber M, Horta H. No road is long with good company. What factors affect Ph.D. student's satisfaction with their supervisor? High Educ Eval Dev. 2021;15: 2–18. doi:10.1108/HEED-10-2020-0044
44. Bekova S, Gruzdev I, Dzhafarova Z, Maloshonok N, Terentev E. The portrait of Russian doctoral student. Contemporary Education Analytics. 2017. Available: https://ioe.hse.ru/data/2017/12/06/1161555067/CAO_7(15)_электронный.pdf
45. Bekova S. Does employment during doctoral training reduce the PhD completion rate? Stud High Educ. 2021;46: 1068–1080. doi:10.1080/03075079.2019.1672648
46. Offerman M. Profile of the nontraditional doctoral degree student. New Dir Adult Contin Educ. 2011;2011: 21–30. doi:10.1002/ace.397
47. Zhuchkova S, Terentev E. Non-linear path to a doctorate: a comparison of direct- and indirect-pathway doctoral students at Russian universities. High Educ. 2024;87: 1729–1747. doi:10.1007/s10734-023-01087-9
48. Zhuchkova S, Bekova S. Building a strong foundation: How pre-doctorate experience shapes doctoral student outcomes. González Brambila CN, editor. PLOS ONE. 2023;18: e0291448. doi:10.1371/journal.pone.0291448
49. Kumar V, Wald N, editors. Global Perspectives on Enhancing Doctoral Co-Supervision. Singapore: Springer Nature Singapore; 2024. doi:10.1007/978-981-97-0460-6

Click here to download Data Review URL

https://doi.org/10.7910/DVN/YKNXO3
