# Supplementary material for: Choosing to succeed? Insights into doctoral students’ supervisor selection and its outcomes
Source: PLoS One. 2025 Jul 21;20(7):e0328471. doi: 10.1371/journal.pone.0328471 (PMC12279141; doi:10.1371/journal.pone.0328471)
Supplement: Attachment — Submitted filename: Response to Reviewers.docx [file pone.0328471.s003.docx]

Dear Editor,

We sincerely appreciate the valuable comments provided by the reviewers. We have carefully reviewed each comment, and we believe that their insightful feedback has greatly enhanced the revised version of our manuscript. Our detailed responses to each point can be found in the table below.

| **Reviewer’s comment** | **Author’s response** |
| --- | --- |
| **Reviewer #1** | |
| *Overarching comments* | |
| Structure - please improve the structure by arranging your headings well. Presenting the section of data before materials and methods confuses the reader; combing the results and methods; and discussion with conclusion also confuses the reader. | We changed the structure of the manuscript. In particular, we merged the “Data” and “Materials and methods” sections into a single “Methods” section to create a more coherent framework for describing our procedures. Within this section, we re-labeled the “Data” as “Sample” for greater clarity in presenting our participants and data collection. We also separated the “Discussion” and “Conclusion” sections. |
| Statistics - both the introduction and literature review could benefit a lot from additional statistics that relate to those who chose supervisors themselves, did they complete timeously or with a struggle, or those who were compelled to work with a particular supervisor, did they complete timeously, or did they have a good working relationship. What are the benefits of studying all these selection strategies? this is not clear in the introduction or background. | Unfortunately, after a thorough additional literature review we conclude that there are no available quantitative statistics on this matter. We provide more examples of qualitative studies of various consequences for the selection strategies and more explicitly emphasize the lack of quantitative studies, as well as the limitations of the existing ones. |
| Tables - there are lots of tables that confuses the reader - consider compressing some of the tables. Additional, some of these tables are not referenced on the intext discussion. | We removed two tables from the manuscript that may have been misleading to readers. We also checked the remaining tables that we refer to in the text. |
| Conclusion and recommendation are critical elements for a manuscripts - the current draft requires a separate clear conclusion and recommendations. | We separated the “Discussion” and “Conclusion” sections. Conclusion now only summarises our own results, while “Discussion” provides links with previous literature as well as practical recommendations based on our findings. |
| *Abstract* | |
| Add the conclusion on the abstract | We added a conclusion to the abstract (“The study highlights the critical role of agency in supervisor selection, showing that structured support for informed decision-making – whether through institutional matchmaking systems or student mentoring programs – can mitigate risks of poor supervision outcomes. These findings call for policy interventions that promote transparency in supervisor selection processes, especially in contexts where students have limited choices.”). |
| *Introduction / Literature Review* | |
| L48: what is the significance of studying the relationship between these factors | We added the explanation: “Understanding this linkage is crucial because early-stage selection decisions may either facilitate smooth progress or lead to irreversible conflicts, ultimately impacting completion rates and research quality.” |
| L58: Add a background relating to the benefits of selecting the supervisors, either by the institutional or personal factors | For each of the reviewed criteria to choose a supervisor, we added explanations on how a student can benefit from their interaction. |
| L98: This is a good sentence, consider incorporating the same to the above paragraphs | Thanks for this suggestion. |
| L117: Any statistics about those selecting supervisors for themselves or those who supervisors were chosen for them.  It can be interesting if you can find the existing statistics that shows differences in terms of completion or study progress between those who chose supervisors themselves and those whom the supervisors were chosen for them. | Unfortunately, after a thorough additional literature review we conclude that there are no available quantitative statistics on this matter. We provide more examples of qualitative studies of various consequences for the selection strategies and more explicitly emphasize the lack of quantitative studies, as well as the limitations of the existing ones. Likewise, we cannot provide up-to-date and representative statistics on which is more common in Russia: students choosing their supervisors or their being appointed by the department. However, we can indirectly assert that doctoral students more frequently select their own supervisors, as large-scale surveys indicate that only 16% choose them based on departmental recommendations. We have clarified this point in the text. |
| *Data / Methods* | |
| L155: This arrangement of data before methods is confusing to me as reader - consider incorporating this section to the method section | We changed the structure of the manuscript. In particular, we merged the “Data” and “Materials and methods” sections into a single “Methods” section to create a more coherent framework for describing our procedures. Within this section, we re-labeled the “Data” as “Sample” for greater clarity in presenting our participants and data collection. |
| L156: Please unpack the survey - how was it like? Has the survey been tested for reliability and validity? What type of questions were included in the survey. | We significantly extended our description of the survey. |
| L243: Where are you referring or narrating this table | We decided to remove this table and this part of our paper as non-relevant for our research. |
| *Conclusion / Discussion* | |
| L345: What conclusion can you draw based on your findings? what advice/s can you give to institutions regarding doctoral supervisor selection? Can institution change their current practice?  Separate conclusion from discussion. make recommendations based on your findings.  The article structure require major adjustments. I struggle to follow when reading the article. | We separated the “Discussion” and “Conclusion” sections. Conclusion now only summarises our own results, while “Discussion” provides links with previous literature as well as practical recommendations based on our findings. We also extended this part of the article. |
| L348: what is your conclusion based on this statement? | We included main conclusions after each finding. |
| L412: Which reference style is practiced here? Be consistent as well | We checked the References section for consistency with the style of the journal and corrected inaccuracies. |
| **Reviewer #2** | |
| *Overarching comments* | |
| The manuscript needs a thorough review and edit for English language. For example, authors switch from plural to singular language but should stay plural. | We relied on formal proofreading procedures in the new version of the manuscript. |
| Structure of sections is quite odd, - the distinction between the Methods and Results is unclear. | We significantly restructured our methodological section of the manuscript, as well as removed irrelevant descriptive results from this section. |
| Methods section is not organized into traditional subsections such as Sample and Participants, Procedure, Measures, and Analysis. | We changed the structure of the manuscript. In particular, we merged the Data and Materials and methods sections into a single Methods section to create a more coherent framework for describing our procedures. This section is now divided into more traditional Sample, Measures, Analysis subsections. |
| Unclear if the study is about "Russian doctoral students" or "doctoral students in Russia", the latter which would include foreign born students in Russia. | We refined the text for clarity. Our study encompasses doctoral students educated in Russia, including those who were born outside the country. |
| *Abstract* | |
| L11: "Research usually..." - which research? | We clarified in the manuscript that the discussion pertains to the field of doctoral supervision. |
| *Introduction* | |
| L36: Add citations to back up argument. | We added citations to support this argument. |
| L40: "An inappropriate" - for clarity of meaning, probably better to say "unsuitable" or "unfitting". | We made the suggested replacement. |
| L46: "..demographic factors" - give examples. | We included examples of demographic factors in the Introduction. |
| L50-51: "..various selection strategies contribute to the difficulties during doctoral training" - hard to follow argument, give examples. | We clarified in the text that this refers to difficulties in interacting with the supervisor. |
| L78: After "Doctoral students" add "usually" before "evaluate". | We added the suggested word. |
| L85-87: This appears quite serious and would warrant more elaboration. | We added more discussion and references on this matter. |
| L89: "...supervisor's background... likely explained" - please add citations to back up argument. | We added citations to support this argument. |
| L97: "These papers" - revise to "These studies". | We made the suggested replacement. |
| L98: "..these factors can vary", Which factors and vary how? Clarify meaning. | We revised this part of the paragraph to clarify its meaning. |
| L107: "..generalizing the obtained findings" - in Russia? Elsewhere? Your study is exclusive to Russia. How does that move the literature forward? | We clarified some features of our study that may improve literature in this part of text. In particular, we shifted our focus from generalization to the fact that we present a larger sample from a nationwide survey that covers different categories of doctoral students and different organizations and allows us to conduct quantitative analysis of several factors simultaneously. We additionally added how our study could be useful to policy-makers. |
| L115: "More than half of Russian doctoral students are male (66%)..". To be exact, this is 2/3. | We don’t use these exact statistics in the updated version of the paper (see next comment for details). |
| L115: Note that the argument about 2/3 of Russian doctoral students being male and that the sample employed in this study is 53% female seriously calls to question the generalizability of the findings if unaddressed. | In our original draft, we presented the last and actual figures for the 2022-2023 study year. However, because our survey sample was collected at the end of the 2021-2022 academic year (May-August 2022), when the proportion of male doctoral students across all types of organisations was 56%, we recognized the potential for confusion. To maintain consistency in our statistical reporting, we therefore used data from the 2021–2022 academic year and substantiated these figures by citing an authoritative statistical compendium. Additionally, we extended our description of limitations to warn readers on potential biases produced by overrepresentation of females. |
| L117-119: Please give actual numbers. Statements about "challenges" in Russian doctoral education are unverified if left without actual trend numbers. For instance, what is the "crisis of Russian doctoral education"? | We provided relevant statistical data to this paragraph. |
| L123: "..supervisors in Russia are still the main figures in a doctoral journey.." - as opposed to what? Please clarify meaning. | We clarified this part (“supervisors in Russia are still the main figures in a doctoral journey, i.e., they still provide all the guidelines on how to prepare and defend dissertation as opposed to the structured models where some of the necessary knowledge could be provided by doctoral school or other faculty”). |
| L127-128: "..practices of team supervision" and "not regulated normatively" - please give examples for clarity. | We clarified this part (“practices of team supervision (i.e., cases when a student has several official supervisors) are rare [37] and can only be possible in circumstances of multidisciplinary research or on programs arranged by two institutions [38]”). |
| L133-134: "..high academic requirements... 3 papers in highly ranked journals" - is this a requirement to defend a dissertation or some other milestone? Please clarify. | We clarified this part (“to get to a dissertation defence”). |
| L136-139: Please explain context. Doctoral programs and procedures differ between places and countries. These are for example not the traditional processes in the US. Making sure that all audiences understand your narrative is important. | We tried to clarify the specifics of the Russian context in this part: “Thus, defences in Russia follow strict formal requirements mandated by the Higher Attestation Commission, including mandatory pre-defence procedures (which require departmental reviews, approval timelines, formal announcements on the websites), document verification at multiple administrative levels, standardized formatting rules for dissertation manuscripts, etc. Usually it is supervisors who navigate these bureaucratic flows, i.e., interpret these requirements for candidates, coordinate with dissertation councils, as well as secure necessary approvals.” |
| L145-146: Clarify meaning. Can you not become a supervisor if you are hired more than 30 days after each student's enrollment? | We clarified this part (“it is obligatory for each student to choose and approve a supervisor within one month after the start of the program”). |
| *Data* | |
| This is where I would think the "Methods" section should begin. | We merged the *Data* and *Materials and methods* sections into a single *Methods* section to create a more coherent framework for describing our procedures. Within this section, we re-labeled the *Data* subsection as *Sample* for greater clarity in presenting our participants and data collection. |
| L170: 2392 were in the numerator but the denominator is not included. Please clarify and insert a response rate. | The survey employed a quota‐based sampling approach (this information, along with the established quotas, is detailed in the “Sample” subsection). Invitations were distributed in a manner that prevented determining the exact number of individuals who received or viewed the invitation link (university administrators were responsible for that, not the authors of the paper). Because a precise denominator is unavailable, a response rate cannot be computed and is not applicable to our data.  To enhance the transparency of our research and the characteristics of our data, we have incorporated an improved description of the survey data and collection methods in the manuscript. Additionally, the revised manuscript notes that the quotas were based solely on formal characteristics rather than on demographic characteristics (e.g., gender, age). Given this approach and the potential for bias, we emphasize that our study explores relationships rather than drawing population-level conclusions – a point further elaborated in the Limitations section.  We also would like to emphasize that, giving all the limitations, the MEMO data used in our research remains the only up-to-date survey data covering the all-Russian sample of doctoral students. |
| L171: Why are students from "research institutes" excluded? Unsubstantiated to cut 15% of the sample in this way. | We included explanation of removal this subgroups from the analysis related to the specifics in doctoral education environment in research institutes in Russia (including less formal relationships between supervisors and doctoral students, higher level of inbreeding among doctoral students and lower variety of career trajectories, less structured doctoral programs, more active integration of students into real research projects). |
| L183: 53% of the sample being female while 66% of doctoral students are male seriously calls to question the generalizability of the sample. | According to federal statistics for the period corresponding to our data collection process, 56% of doctoral students were male. We have updated the manuscript to reflect this correction (see previous responses for details).  But even now we observe over-representation of women in our sample. In our response above, we provided additional details about the survey and noted that demographic characteristics were not subject to quotas in the nationwide survey. However, our primary objective was to explore relationships rather than to generalize our findings to the entire population of doctoral students in Russia. Accordingly, we addressed these potential biases in the Limitations section. In particular, we reiterated the need for caution when interpreting the results in terms of generalization, given that the sample is non-probabilistic and based solely on formal characteristics quotas.  We also would like to emphasize that, giving all the limitations, the MEMO data used in our research remains the only up-to-date survey data covering the all-Russian sample of doctoral students. |
| L184: with most participants being 1st or 2nd year, - how many of those have not yet selected a supervisor? Or if all already do than that should be clarified. | We clarified that at this point supervisors are already assigned to students according to the national regulations. |
| *Materials and Methods* | |
| L188: Materials and Methods - this section reads like a "Measures" subsection. | We renamed the subsection to Measures within the Methods section, as suggested, and introduced a three-level heading structure to enhance navigability and clarity. Specifically, we created the following subsections within Measures:   - *Selection strategies*, where we describe our main variables of interest, - *Difficulties in interaction with a supervisor,* which addresses our first dependent variable, - *A student’s lack of confidence about defence*, detailing the manipulation for our second dependent variable, and - *Control variables*, describing the additional variables included in the analysis. |
| L202: What about "other reasons" - was there no option for that? | This option was included in the questionnaire, but we excluded it from the analysis because the category contained very few observations and the responses within it were highly heterogeneous. This is now clarified in the text. |
| L206: As far as I can tell, these are analytical results and should belong in the Results section. | These are descriptive statistics of our variables, not the analytical results. At the same time, we shortened this part by integrating the percentages into the description of options. |
| L210-211: Unclear how PCA accounts for variations in response numbers. With multiple choice questions some students may have selected 1 option and some all options. Please clarify. | Different number of selected options should not be an issue for this part of analysis as we use dummy variables representing all the provided options. Students who select only one option have, let’s say, a distribution 1 0 0 0 0 0 0 0 (if he or she chooses the first option), those who select all the options have a distribution 1 1 1 1 1 1 1 1. As long as the length of these vectors are equal, we can use these set of variables in analysis. |
| L223: Briefly expplain the Thurstone method. | We added a brief explanation of the Thurstone method and supported this short description with the following citation |
| L223-224: "These scores.." - which scores? | We inserted a word that clarifies the meaning. |
| Table 3: Findings have unclear relevance to the study. Please clarify in the Introduction why these findings are important and part of the study. | We excluded this table from the manuscript. |
| Table 3: Last section in analysis. F-statistic assumes linear trends but the "Field of Study" is nominal variable. This analysis appears incorrect. | We excluded this table from the manuscript. |
| L252-253: "N=1606 excluding..". Unclear relevance and meaning. | We changed the wording to make it clear that it is about missing values (who had difficulty answering this question). |
| L285: "A significant proportion" - unclear meaning. What is a "significant proportion". | We replaced this phrase with more precise wording. |
| *Results* | |
| L304: By here a lot of "Results" have already been shown. | We have moved the description of all analytic results, previously included in the Methods section, to the Results section. This change also applies to the description of the distribution of responses. |
| Tables 5 and 6: Why so many models and why this order of variables in the models? Please clarify. | We clarified that “Each set contains several models, into which we sequentially incorporated predictors and control variables described in the previous subsection. We rely on this technique to ensure that the effects of our variables of interest are stable and do not disappear when considering other important factors.”, as well as “In the regression tables, we firstly present our variables of interest, and then all the control variables (starting from the substantial ones to more formal ones).”. |
| **Journal Requirements** | |
| 1. Please ensure that your manuscript meets PLOS ONE's style requirements, including those for file naming. The PLOS ONE style templates can be found at  https://journals.plos.org/plosone/s/file?id=wjVg/PLOSOne_formatting_sample_main_body.pdf and  <https://journals.plos.org/plosone/s/file?id=ba62/PLOSOne_formatting_sample_title_authors_affiliations.pdf> | We arranged the manuscript in accordance with the requirements of the journal. |
| 2. We noted in your submission details that a portion of your manuscript may have been presented or published elsewhere. “We have published regression dataset that used in our research at Harvard Dataverse under CC0 v1. https://doi.org/10.7910/DVN/YKNXO3”  Please clarify whether this [conference proceeding or publication] was peer-reviewed and formally published. If this work was previously peer-reviewed and published, in the cover letter please provide the reason that this work does not constitute dual publication and should be included in the current manuscript. | This work was not peer-reviewed and published previously. The mentioned link refers to a dataset that we used in the analysis and made publicly available according to the PlosONE policy. |
| 3. Please include your full ethics statement in the ‘Methods’ section of your manuscript file. In your statement, please include the full name of the IRB or ethics committee who approved or waived your study, as well as whether or not you obtained informed written or verbal consent. If consent was waived for your study, please include this information in your statement as well. | We included this information in the “Methods” section. |
